# Integration and completion: life wisdom and happiness of Chinese older adults from a life course perspective

**DOI:** 10.3389/fpubh.2025.1575099

**Published:** 2025-05-16

**Authors:** Sheng-Li Cheng, Xin Zhang, Shushan Liu, Chenxu Zhao, Sizhen Bi

**Affiliations:** ^1^School of Philosophy and Social Development, Shandong University, Jinan, China; ^2^Bussiness School, Shandong Agriculture and Engineering University, Jinan, China

**Keywords:** life courses, Chinese older adults, life wisdom, happiness, qualitative research

## Abstract

**Introduction:**

Life course theory posits that historical events that transpired in the past shape an individual’s well-being. In the last century, China experienced a series of significant events in rapid succession, including the Anti-Japanese War (AJW), the Civil War (CW), the Great Famine (GF), the Cultural Revolution (CR), and the Reform and Opening of China (ROC). These early life experiences may have lasting effects on older adults. Concurrently, given the discrepancies in life course, there may be discrepancies in well-being and perceptions of life between Chinese and Western older adults.

**Methods:**

A qualitative study was conducted within a constructivist paradigm. In-depth interviews were carried out with 16 older adults using a standardized inventory. A theoretical framework was constructed through the effects of temporary breakdown on the life course and “vital involvement in the necessary disinvolvements of old age (VINDOA).” Data were coded using the Nvivo-12 software.

**Results:**

The themes of well-being and life wisdom among Chinese older adults centered on (1) valuing education, strong family ties, and active social participation, which were associated with personal experiences of well-being. (2) Satisfaction and gratitude for social welfare, being happy and worried about current social situation in the same time, which are related to older adults’ values of society. (3) Expectations, encouragement, and transmission of wisdom to youth are related to their views of young people and youthfulness.

**Conclusion and discussion:**

A key finding is that how older adults perceive life strongly influences their present experiences and sense of well-being. In addition, the social class in which older people live plays an important role in the connection between a challenging past and a fulfilling present. Furthermore, social development has a positive impact on the well-being of older adults, while the level of social participation of older adult people exerts a further influence on their own well-being and their capacity to contribute to society.

## 1 Introduction

The concept of the “life course” is used to analyze the process of change in human life, with a focus on the time, context, and historical processes that shape an individual’s life from birth to death. In Western life course research, a common question is how a changing society affects the development of individuals. Elder (1) emphasizes the impact of social forces on life outcomes and the shaping of individual trajectories. As life course theory has evolved, increasing attention has been paid to the interplay between social pathways and both historical and developmental contexts (2). Mastery of the past life course is necessary to gain a deeper understanding of an individual’s later years. Role histories, shaped by past events, influence health, adjustment, and well-being in later life (2–4).

Erikson’s life cycle theory outlines eight stages of psychosocial development, with the final stage being older adulthood. In this stage, individuals are tasked with reflecting on their past while staying engaged in the present (5–7). Re-experiencing past events is a key psychological task at this stage (8, 9). In *The Life Cycle Completed*, Erikson defines “wisdom” as the strength gained in the struggle between integration and despair. In *Vital Involvement in Old Age*, he states more directly that integration and despair are two diametrically opposed tensions that the individual will face in old age. Furthermore, the individual must strive to achieve a balance between these two opposing forces, integrating each psychosocial theme, and fostering a sense of integration as the dominant experience during old age. Additionally, the pursuit of “wisdom” and maintaining “the vital involvement in the necessary disinvolvements of old age (VINDOA)” is essential for meaningful aging (6, 9).

The successful aging perspective (SAP) is a widely used concept in western research on older adults’ well-being. However, Stowe and Cooney (10) criticized it for neglecting cultural and historical context, as well as the influence of social relationships and structures. They proposed rethinking SAP through a life course lens. The life course paradigm (LCP) integrates multiple theories (11–13) and suggests that early life events — such as learned skills, coping strategies, and formative experiences — shape later well-being and life satisfaction (14–16).

In China, both policymakers and scholars increasingly promote the concept of positive aging. Official documents and academic studies now define health not only in physical terms but also as psychological and social adaptation in old age (17–19). Current research on the well-being of the older adults focuses on three aspects: the current status of subjective well-being (20, 21), the causal relationship between different aging modes and subjective well-being (22, 23), and the study of influencing factors (24, 25). Compared to the West, China’s 20th-century historical process was more complex and changeable. Research on the well-being of Chinese older adults who grew up during this period needs to be integrated with the relevant social context. Major events such as the Anti-Japanese War (AJW), Civil War (CW), Great Famine (GW), Cultural Revolution (CR), and the Reform and Opening-up of China (ROC) have influenced the personality traits of citizens during that era. As people navigate life’s challenges, adversity often becomes a source of resilience, reinforcing beliefs such as the idea that “hardship must precede success” (26). Additionally, Da (27) suggests that subjective well-being is a personal evaluation of one’s life satisfaction based on individual standards and social realities. It increases when real-life conditions exceed the set standard and decreases when they fall below it. Also, comparing the current living conditions with the past living conditions is an important criterion for people to measure subjective well-being (28).

In light of the considerations above, we propose the following research question for this paper: from a life course perspective, how do Chinese older adults understand life and what factors shape their well-being in old age? Life course research emphasizes the inter-constructive nature of macro-social structure and micro-individual life. The mutual construction is characterized by the constraints of social structure and the autonomy of individual action (29). By examining the tension between integration and despair in the context of China’s historical transformation, we aim to gain deeper insight into the life wisdom and well-being of Chinese older adults.

## 2 Theorizing life courses

Life course theory originated in the 1920s and has developed significantly over the past century. It offers a perspective for studying individual well-being that begins with significant events and behavioral patterns throughout an individual’s or group’s lifespan. Elder (30) presented four paradigmatic themes of the life course. Firstly, the time and environmental context in which it takes place. An individual’s life course is often epitomized by the events they have experienced over the years of their life, as well as being shaped by those times and events. Secondly, the impact of significant events at different ages. The effects of transitions and events vary depending on when they occur in an individual’s life and on the expectations and beliefs available at that age. Thirdly, the impact of interdependent social relationships on life. Life exists in the interdependence of kinship or other relationships, and socio-historical influences are manifested through this network of shared relationships. Fourthly, the impact of individual choices and actions on life. Individuals are able to construct their life course through their own choices and actions, utilizing the opportunities available to them and overcoming the constraints of historical and social circumstances.

The interviewees experienced various historical events and life transitions, facing difficulties and uncertainties throughout their lives. When comparing older adults in China to those in western countries, differences can be observed in their life plans, stages of purpose, and resulting sense of well-being and happiness. However, it is important to acknowledge that historical events in the western countries have also shaped people’s life trajectories, which can also be used for comparison and analysis.

### 2.1 Different stages of the life course

The life of an average person can be divided into five stages: early childhood, adolescence, adulthood, middle age, and old age (31, 32). Each stage has its own unique characteristics and developmental tasks that drive the socialization process. These tasks include experiencing social roles, building social networks, and assuming social division of labor. Old age is often associated with desocialization, which can lead to poorer health and changes in social roles. However, it can also provide individuals with more free time and opportunities for introspection, both of which can have an impact on their well-being and happiness. The situation in China adds further complexity. Chinese older adults have experienced significant societal changes throughout their lifetime. When they were young, Chinese society was in the early stages of internal and external conflict and regime establishment. Over time, China has developed into a moderately prosperous society. The war, institutional collapse, and disorder have significantly impacted the lives of the population. Individuals experience various challenges and opportunities due to social change and the need to adapt to new social environments and role positions. This is known as the post-transitional life course (33). Individuals who spent their early childhood and adolescence during this era often recount not only their personal experiences and upbringing, but also significant societal events and changes that occurred during this period.

### 2.2 Experiences of well-being within the context of modernity

The post-transition life course comprises two new stages associated with modernity. A living older people may be born in the pre-transition life course expectation or die in the post-transition life course expectation (34). Our respondents experienced both the pre-transition suffering and post-transition prosperity of Chinese society. Experiencing both phases of the life course at the same time resulted in different experiences of well-being for individuals.

Currently, there is little academic attention paid to the question of the well-being experiences expected to result from an individual’s dual life course. On the one hand, a person’s life course changes from pre-transition to post-transition imply that social change is accomplished in a short period of time, which is uncommon in many countries. On the other hand, few studies have focused on the impact of social change as an external factor on an individual’s life course. Through a review of existing literature, this study has found that Sweden’s social transformation can provide valuable insights. Gunnarsson (35) conducted interviews with 20 older adults who were born before the World War II and found that Sweden experienced rapid economic development in the last century, transitioning from a poor country to a welfare state. Most of the interviewees lived in rural areas before moving to cities for education and work as urbanization increased. These senior citizens have witnessed the establishment and growth of the welfare state. The economic development and various welfare policies implemented by the state have provided them with more opportunities and convenience in their lives. The study by Lennartsson et al. (36) found that socioeconomic status in childhood was associated with health in old age, after analyzing the socioeconomic status of the older adults born between 1925 and 1934 and their health outcomes. Additionally, the study found that increases in years of education are directly associated with good health in middle age. The government’s educational policies have facilitated more equitable access to educational resources for citizens. At the same time, these policies have mitigated some of the negative effects of lower socio-economic status on health outcomes.

### 2.3 Changes in well-being resulting from ephemeral breakdown

The outbreak of the World War II caused extensive disruption to people’s lives worldwide, with many young individuals involved in the campaign. Smith et al. (37) conducted semi-structured interviews with World War II veterans and their family members. The study found that the war brought great pain and fear to the soldiers. Only troop camaraderie was a positive emotion that was maintained and continued among inter-service members. After the war ended, many military personnel struggled to reintegrate into civilian life due to traumatic memories. Some military partners and children also experienced domestic violence and grief as a result of the emotional toll on military personnel (37, 38). A longitudinal study by Ardelt et al. (39) confirmed that crises and stresses associated with war may propel resilient service members to achieve higher levels of wisdom and well-being. However, these same experiences may have more negative physical and mental health effects on individuals who are not resilient.

In addition to veterans, the lives and well-being of the general population have been affected to a significant degree. World War II survivors and their family members who experienced a variety of traumas in early childhood and adolescence developed some degree of depressive symptoms and PTSD (40, 41), and the impact of wartime trauma on an individual’s life course is diminished if the trauma occurs at an earlier age (42). Whereas factors such as access to high-quality education, strong family relationships and volunteer work in old age can contribute to achieving a better level of mental health and resilience (41).

The Great Depression of the 1930s disrupted lives in the United States, and the economic status and quality of life of the unemployed population reached its lowest point. The Great Depression resulted in widespread unemployment and social unrest. Workers began to recognize the inherent risks and uncertainties in their careers (43). Additionally, many working-class and unemployed residents were evicted from the city, causing significant harm to their personal interests and well-being (44). However, there were still some positive developments during the Great Depression. Historical statistics show that the physical health of citizens was not adversely affected. Moreover, some studies have found that the experience of economic hardship can promote personal growth and the accumulation of reflective introspection and life wisdom to resolve crises, which can buffer against the negative social effects of economic crises (45, 46).

Regarding the impact of traumatic events, such as wars and economic crises, on individuals, discourse in conventional Western life history research has primarily focused on the effects of ephemeral breakdown in an individual’s otherwise stable and predictable life course, as well as changes in an individual’s physical and psychological well-being (47–49). Most studies on the life course and social change confirm that disruptions to the stable life course often have negative consequences. However, some sociologists have also explored the positive impact that these traumatic experiences may have on individuals, as well as the buffering effect of factors such as education and social networks (41, 50, 51).

In China, the phrase “Yiku Sitian” (contrast past misery with present happiness) has been officially mentioned and advocated, with past suffering serving as a backdrop message to highlight the happiness of the present. During the Cultural Revolution, the “The educated youth (Zhiqing) Going to the Countryside Movement” mobilized young people to move away from urban areas to live and work in remote villages, mines, and factories. However, research has confirmed that this high-pressure environment promoted the development of independent personalities among these youths, which was conducive to improving their adaptability and interpersonal skills (52). The role of Zhiqing’s mentality in labor participation is also significant (53). Many urban youths responded to the CPC and government’s call to proactively experience life in rural and remote areas. According to Wang and Liu (54), the collective memory of the Zhiqing youths serves as a means for a generation to locate and trace their youthful years. They argue that this ‘rose-colored glasses’ review also closely connects the fate of individuals with the history of the country. To this day, China continues to use this spirit of hard struggle as an important method for carrying out ideological and political work with young people (55).

Kahn and Juster (56) define resilience as the speed and completeness of recovery from a crisis event. They confirm that older adults’ resilience buffers their adaptation to stress and challenges. The magnitude and duration of negative events have different impacts on the level of resilience and well-being of the older adults. The duration of the mourning effect is prolonged when faced with events such as the death of a loved one. If these challenges can be anticipated in advance, the resulting negative effects can be moderated. Kahn and Juster refer to these life-course disruptions as “variability”, emphasizing that illness, death, or other crises bring varying degrees of shock and resilience. However, what if we were to consider these events as “permanent”, with the effects of a breakdown lasting from early childhood to old age?

### 2.4 Chinese scholarship on well-being in later life

In recent years, there has been an increasing amount of research linking life course interruptions to well-being in later life. The most commonly studied measure is the level of health in old age. Jiao and Bao (57) argue that childhood is a critical period in the life course and that adverse circumstances in childhood not only have a direct impact on health in adulthood, but also later in life. For instance, the older adults who experienced the Holodomor in childhood had worse health outcomes than those who did not. Moreover, the experience of famine during childhood had a negative impact on education, socialization, and lifestyle (58). These factors, in turn, continued to have a detrimental effect on the health of the older adults (59). Meanwhile, the positive influence exerted by educational attainment on health levels in later life is generally confirmed. Older adults who recall significant youth experiences having lower levels of self-assessment of their health, and years of education as a mediator variable showing a positive correlation with the older adults’ mental health levels (60)

In later life, well-being is composed of a sense of worthiness and happiness. Peng (61) posits that the older adults’ income level increases when they have “Going to the Countryside” experiences. However, their happiness decreases as a result. This may be due to the fact that challenging life experiences hone an individual’s will and have a positive impact on their career. Nevertheless, an individual’s perception of time changes after experiencing these critical moments, and suffering and negative events can cause them to perceive time as longer, leaving behind painful memories. Retrospective life stories can help older adults return to the center of their individual narratives and give full play to their subjective initiative, enhancing their sense of value and quality of life (62).

## 3 Methods

### 3.1 Data collection

This study employed constructivism as its research paradigm and employed qualitative research methods. Sixteen Chinese older adults residing in Jinan City, Shandong Province, were identified for interviews through purposive sampling. The rationale for selecting Jinan as the study site has been previously described in detail in a related study (34). The participants included both Jinan local residents and those who were born in rural areas and later went to school or worked and settled in Jinan, as well as those who followed their children and relocated to Jinan when they were old. During the participant recruitment process, the research team conducted field visits to various public venues across different districts and counties of Jinan City, such as community centers, parks, and senior activity centers. By engaging older adult individuals in casual conversations and building initial trust, the researchers invited those who met the study’s inclusion criteria to participate in the interviews. After the initial contact, the purpose, content, and procedures of the study, as well as the voluntary nature of participation and confidentiality measures, were thoroughly explained to the potential participants. Formal interviews were conducted only after obtaining informed consent.

At the end of 2012, the research team conducted semi-structured interviews with 16 participants, each lasting approximately 1–2 h. The interviews encompassed a range of topics, including memories of different life stages, current living conditions, outlook on future life, wisdom transmission and sharing, and other aspects. In preparation for the interviews, the research team developed a formal interview outline, which the interviewer could use with relative flexibility during the interviews to ensure that the interviewed people could tell their life stories without restrictions. In this study, the interview outline was developed under the concept of standardized biography, whereas the vein of sharing the older adults’ life stories was non-standardized (34, 63).

The research team adhered to ethical standards. First, the staff members who determined the list of interviewees were not acquainted with the interviewees, ensuring the relative independence of the sampling and interviewing process. Second, the older adults selected for the interview had the final say on whether or not to be interviewed. If someone declined, the interviewer would establish contact with the next people to be interviewed. Finally, prior to the commencement of the interviews, participants were clearly informed that all data would be utilized for academic purposes only and that all personal information disclosed would be anonymized.

The interviewed people were born between the late 1930s and early 1950s and were aged 61 to 75 at the time of the interviews. Having experienced both the devastation of war and the scarcity of food in their early years, as well as the bountiful period of national wealth and prosperity in their later years, these two stages are associated with modernity and are consistent with the characteristics of life course of the post-transition period. The gender ratio and urban–rural distribution of the interviewees are relatively balanced, with 8 men and 8 women each, and 8 urban and 8 rural residents each. The data from the interviews is presented in Table 1.

**Table 1:** Characteristics of the study participants.

No.	Gender	Age	Year of birth	Region	Occupation
1	Female	63	1949	Rural	Accountant
2	Female	73	1939	Urban	University Teacher
3	Male	64	1948	Urban	Worker
4	Male	71	1941	Rural	Educator
5	Male	71	1941	Rural	Farmer
6	Female	70	1942	Urban	Worker
7	Female	63	1949	Urban	Accountant
8	Male	74	1938	Rural	Professor
9	Male	75	1937	Rural	Teacher
10	Male	66	1946	Rural	Farmer
11	Male	72	1940	Urban	Farmer
12	Female	74	1938	Rural	Nurse
13	Female	61	1951	Urban	Worker
14	Female	74	1938	Urban	Civil Servant
15	Male	72	1940	Rural	Worker
16	Female	74	1938	Urban	Doctor

### 3.2 Data analysis

**Figure 1:**
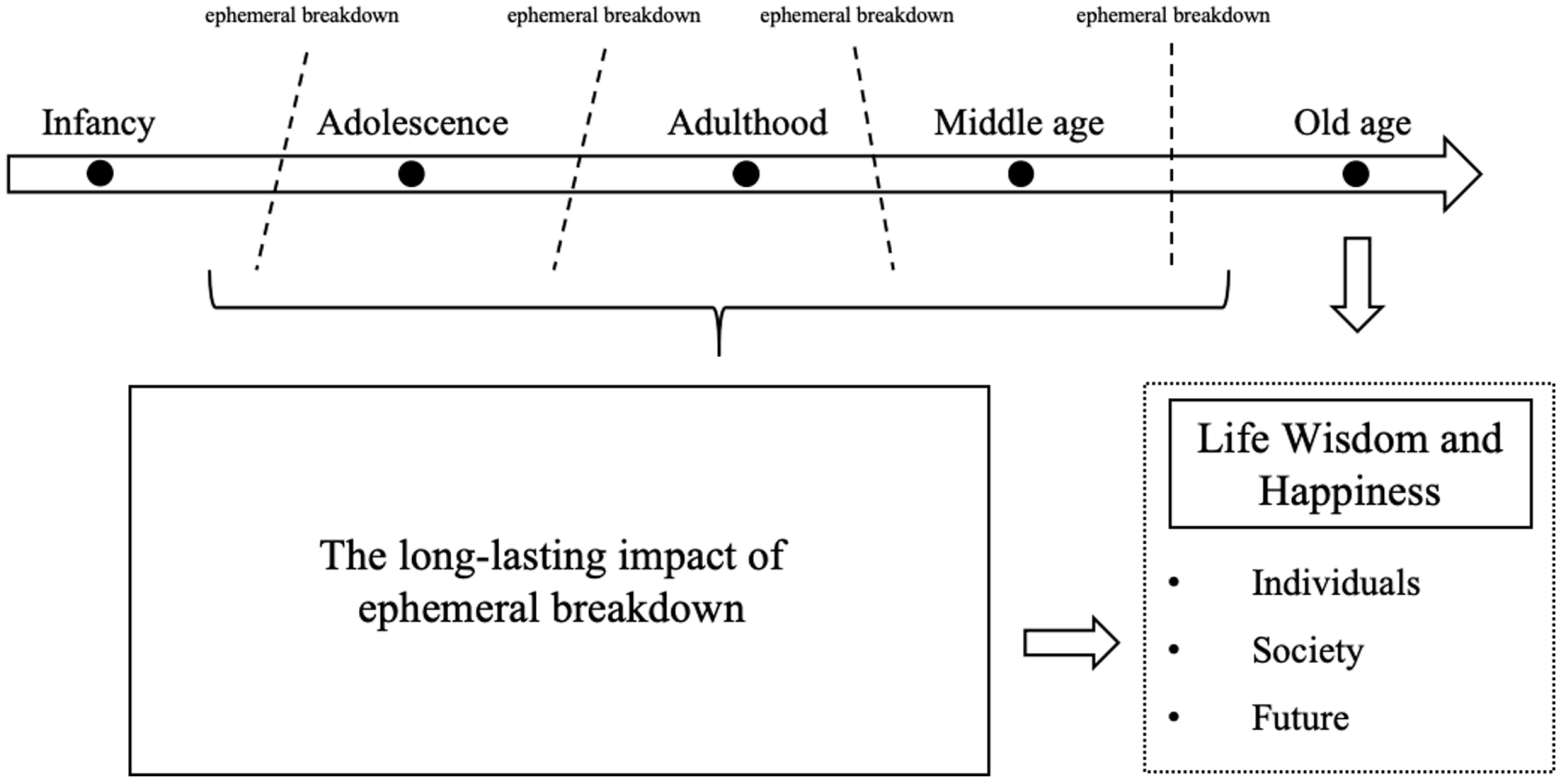
Theoretical analysis framework.

The older adults had experienced significant challenges throughout their lives, including the AJW, CW, GF, and CR. These events had a profound impact on their life course, resulting in long-lasting effects (34). Concurrently, they are at the end of the life course, the stage of old age. During this stage, older adults must overcome despair and fear in order to achieve integration and completion. This process leads to the realization of life-cycle harmony and unity (64). The pervasive influence of ephemeral breakdown on the life course and the phenomenon of VINDOA are intricately intertwined, collectively shaping the invaluable life wisdom of the Chinese older adults and the sense of well-being that accompanies it. This sense of well-being is presented in three dimensions: individual, social, and future-oriented. The theoretical framework established by the study is shown in Figure 1.

The study imported 16 interviews into the Nvivo-12 and coded them according to these three dimensions. This resulted in 3 themes, 8 sub-themes, 30 initial concepts, and 256 informational reference points. These included perceptions of life, perceptions of society, and expectations for younger generations. Subsequently, the study undertook a process of sorting and refining the intrinsic logical relationships among the three levels of coding. This was followed by the identification of the content and vein of what is included in the life wisdom and well-being of Chinese older people from a life course perspective.

## 4 Results

### 4.1 A preliminary analysis

The majority of the interviewees expressed contentment with their current state of life, demonstrated strong familial ties, and exhibited a profound sense of responsibility towards their dependents. They were all born into large families with four to five siblings, and are now starting their own families, with the majority having three or four children. This aligns with the original familial structure. Prior to retirement, the majority of respondents were “unit people,” a term derived from China’s planned economy. This designation signifies that the unit is responsible for the life, death, and sickness of its employees. Consequently, it is challenging for them to transition to alternative employment opportunities during their working years. Following retirement, these individuals exhibit a high degree of autonomy, focusing their daily lives on the care of their grandchildren, exercise, and the cultivation of their own hobbies. They are satisfied with the protection and services provided by the state.

The challenging experiences of their early lives have imbued the lives of Chinese older people with a distinctive depth of meaning. The profound changes that have occurred in the context of the times have intensified their understanding of their current lives. The objective reality of societal prosperity and progress is evident, and the contrast between the present and the past has heightened the subjective sense of happiness experienced by the Chinese older adults. The majority of the interviewees’ later lives are characterized by an integration of their past experiences with their current circumstances, resulting in the formation of their distinctive wisdom. This wisdom is not only manifested in the realization and fulfillment of their personal lives but also pervades their comprehension of social development and their expectations for the growth of younger generations.

### 4.2 Experiences of personal well-being: insights into the lives of older Chinese people and the path to happiness

Our interviewees have experienced 70 years of upheaval, change, and development. Three important themes—education, family, and social participation—run through their life stories. The overwhelming majority of our interviewees consider themselves to be happy. Their assessment of happiness is not only based on absolute and objective criteria, but also on their past life experiences, which have been gradually transformed into their unique wisdom through lifelong of experience and comparisons between the present and the past.

#### 4.2.1 Education

The interruption of education for the majority of the interviewees was a consequence of the war and the CR. The poverty of social conditions and the breakdown of education system by the social environment also made continuous education very difficult. However, the interviewees who lived through those years (e.g., interviewee 1, 2, 4, 10, 13) cherished the opportunity of education more and became more self-reliant as a result.

##### 4.2.1.1 Disruption of education, self-empowerment, and valuing education

The war resulted in the disruption of education for many individuals at the time. The majority of the interviewees who were of school age at the time were unable to receive an education due to social unrest. The subsequent CR resulted in the collapse of the education system, creating a profound memory of interrupted education for many interviewees. At the same time, this experience was perceived as a regret, as it prevented them from pursuing higher education and realizing their life aspirations.

*“Actually, I did not go to elementary school for very long time, because we fled as a refugee when the* AJW *started.”(No. 2, born 1939)*

*“The CR started in 1966. Students didn’t have classes then, schools were closed, and no class for us to attend. Until the CR ended, people like me started to have chance to go to high school by recommendation.”(No. 1, born 1949)*

The older adults, particularly those who had received an education, attached great importance to education, having experienced the scarcity of educational resources themselves. As No. 3, born in 1948, stated, *“In my memory, my father was strict to us, always telling us to study hard. At that time, we went to school all by ourselves, and the conditions were not as good as they are today”*.

Despite the challenging circumstances of their education, the interviewees held a positive outlook, cherishing the educational opportunities they had obtained. For the older adults, the ability to access education despite the difficulties they faced kept them in a positive frame of mind. As No. 3 stated, *“Although life was hard then, our mental outlook is very good, very positive, there is no complaining and dragging on”.*

As a consequence of fleeing from the war, No. 2 received almost no primary education. During the CR, she endured significant torture and suppression, and also observed the suffering of her mother and teachers. These experiences have reinforced her conviction that education can facilitate the transformation of one’s destiny, as it can provide a means of making an impact and protecting oneself and one’s family in times of crisis.

*“At that time, you needed to study hard if you wanted to stand out of people and survived in hard world. You could only change your fate by going to university…” (No. 2, born 1939)*

#### 4.2.2 The inter-connectivity between family members

Most of the interviewees had experienced family breakups and displacement of relatives due to social instability such as wars. The Chinese people have long valued the concept of the family, and family breakups have had a profound impact on them, which also affects their subsequent values and education of their offspring, emphasizing that respect for parents and solidarity between siblings have become important themes in their lives.

##### 4.2.2.1 The dissolution of families, the reverence for parents, and the assistance of siblings

When the interviewees were still in their formative years, they were particularly vulnerable and in need of the nurturing and support of their families. However, during that period, the war-torn and poverty-stricken society brought them a childhood full of suffering and sadness. Some of the elders discussed how their displacement from their homes during their early years and their loss of contact with important family members had caused many childhood deficiencies. Accordingly, in the accounts of the interviewees, their childhood experiences were invariably shadowed by the anguish and helplessness engendered by transient crises such as the loss of loved ones and the dissolution of families.

*“I couldn't get my father's care when I was a young child. When I was two years old, when the AJW was going on, my father went to join the People’s liberation army to fight Japanese imperialism.” (No 9, born 1937).*

*“I never saw my father and didn’t know what he looked like. Before I was born, my father went to join the army and became a spy of the CPC. My father was the only son of my grandfather and was also the youngest among his siblings.” (No 12, born 1938)*

The experiences of childhood displacement and the absence of a complete family unit have led to a heightened appreciation for familial love. The accounts of the interviewees revealed a profound reflection of the traditional Chinese concept of filial piety, with the majority of them assuming the obligation of supporting their parents to the fullest extent possible and demonstrating a strong sense of responsibility towards their families.

*“I took care of my mother for 13 years before she moved to my young sister’s apartment after my brother in law (his sister’s husband) died because their apartment was larger.”(No 16, born 1938)*

*“My grandmother was not healthy when she was old. My sister and I took care of our grandmother in turn.”(No 6, born 1941)*

Similarly, the interviewees demonstrated a profound focus on sibling solidarity and mutual assistance, which may be related to the significant turbulence and family breakups they experienced in their early years. The majority of the interviewees resided in families comprising numerous siblings. However, they exhibited a remarkably unified and amicable relationship with one another, providing mutual assistance, exhibiting a tendency to prioritize self-interest over kinship, and instilling in each other the motivation to live a fulfilling life in the present due to their shared experiences of adversity.

*“I felt most important person for me was my elder cousins, because my family was very poor at that time, my female elder cousin worked in a factory and my male elder cousin worked at accounting college, they often aided my family, and I also went to their home.” (No. 3, born 1948)*

*“I am the oldest child in my family, and have several brothers and sisters. I felt responsibility to be the oldest brother to take good care of young brother and sisters. Being the oldest brothers I should let them eat first when there were good foods, do work first when there are farm work in field. That is the responsibility which the oldest brother should take.” (No. 10, born 1946)*

#### 4.2.3 Active social participation

Social participation can be defined as the role-playing and involvement of participants in social interactions with the objective of achieving resource sharing and the fulfillment of personal needs within society (65). The interviewees, from their early years to their current age, have experienced and witnessed the development and progress of society. In comparison to their previous poverty and suffering, they now enjoy a happy life, which fills them with gratitude and contentment towards society and their country. Consequently, they also wish to participate in society, integrate into the collective, and dedicate themselves to the collective. Moreover, for our interviewees, the majority of their early life experiences were characterized by sadness and pain. However, these experiences have instilled in them a resilience that enables them to navigate life’s challenges, including physical deterioration. Instead of being overwhelmed by these difficulties, they have adopted a more optimistic outlook, leading to a more fulfilling and colorful life in the present.

##### 4.2.3.1 Gratitude to society, recreational life, and utilizing one’s spare time at work

As they recounted, the previous life was not only constrained by harsh conditions but also lacked autonomy. They described a sense of confinement in their work and life, with their work being assigned to them, leaving little time for personal interests. The contrast between the living conditions and environments of the past and the present led our interviewees to express feelings of contentment and gratitude for the present society.

*“In the past, people couldn’t follow their interest to choose their job, but now people can follow their interest to choose job, you can freely develop your interest, you can search and try to find what you like to do.” (No. 8, born 1938)*

*“You don’t know how comfortable life you are living. When I was as young as you are now, it was very difficult for me to have money to spend. There was little money or no money, no good food to be eaten.” (No. 1, born 1949)*

Additionally, the majority of the interviewees expressed a sense of optimism and positivity regarding their future senior years. For instance, No. 14 and No. 15 aspire to enrich their daily lives through extensive participation in community activities. Their future plans prioritize physical health, with even exercise becoming a form of social participation and socialization.

*“I do exercise every day now, and I am healthier than before. I come to this little forest square here in Shandong University to do exercise every morning.” (No. 15, born 1940)*

*“I put the papers with words on trees and sang these songs together with other old people.” (No. 14, born 1938)*

Nevertheless, some respondents, having endured a challenging early life, expressed discomfort with the prospect of a comfortable retirement. They were not accustomed to the sudden ease and leisure that came with retirement. Many respondents also expressed a desire to continue working in order to utilize their spare time effectively and maximize their value in their later years, thereby enriching their lives.

*“I felt I was not old enough and want to do something then. Later I continually run a small dining table program which serves food for students who do not go back home to have lunch or dinner for about 6-7 years.” (No. 13, born 1951)*

*“I felt a sense of loss sometimes after I retired, I felt I wasn’t done, I was only 55 then.” (No. 15, born 1940)*

### 4.3 Perceptions of society: social changes and values from the perspective of older people

In discussing their perceptions of contemporary society, the older adults (e.g., Nos. 4, 5, 7, 9, 10, 11, 13, and 16) employed similar evaluative criteria, and their perceptions of contemporary society encompassed both positive and negative aspects, as well as concerns related to social issues and social change.

#### 4.3.1 Contentment and gratitude

The older adults, having experienced economic development and social progress, are now able to enjoy a contented and happy life. No. 1 said: *“I had never dreamed this kind of life before. I believed that life would be better and better because of social policies we have.”* This older woman, born in 1949, experienced GF and CR despite the war having ended before her birth. During the period of poverty, hunger, and chaos that followed, she contemplated that life would never improve. Consequently, she is profoundly grateful for the present life and its opportunities.

##### 4.3.1.1 Social participation, social security, and services

From their narratives, it becomes evident that the state and society have provided them with the requisite social security and services, including financial support and medical care in their later years, as well as community cultural construction and the improvement of public infrastructure in their daily lives. In their past life of suffering, they seldom envisioned happiness after difficulties, and now these guarantees and services have become a consistent and important criterion for them to evaluate today’s society.

*“Every aspects of my life were quite good, you see, my pension rise a lot recently, my life was quite good*…*, I realized what I wanted. I never dreamed I could have today’s good life. How dared I dream for this kind life before; I had never dreamed this kind of life before. I believed that life will be better and better because of social policies we have.”(No. 1, born 1949)*

*“I am very satisfied with my life now. The state gives me life subsidy every month, and I live here where I can get care needed; I can get reimbursement when I go to see doctor and pay medical fee.” (No. 10, born 1946)*

The views of individuals on societal issues are shaped by their past and present life experiences. Those who have experienced poverty, hunger, and war often express concern about the economy and the challenges of securing their livelihoods in retirement. As age, older adults tend to prioritize the protection that society can provide, particularly in light of the decline in their physical abilities. The provision of pensions, especially medical insurance, and the steady rise in the level of protection have undoubtedly increased the satisfaction of the older adults with today’s society and the degree of happiness in their old age.

*“The state gives me 4000 yuan per month as pension. I told my wife that ‘socialist system is really good’. People are easier to be sick and should pay more medical fee when they are old, but we were covered by social medical insurance.”(No. 9, born 1937)*

*“I am satisfied about my whole life with nothing to be regretted…I was covered by social security system after I retired, such as social medical insurance.” (No. 7, born 1949)*

Despite having withdrawn from the workforce, our interviewees continue to engage in social activities (e.g., Nos. 2, 3, 4, 7, 10, 14, 15, and 16). They enrich and integrate their later life through exercise, recreation, and study. The cultural development of the community and the development of public service facilities have provided them with more opportunities to continue to build their social networks, which helps to achieve a balance between a sense of despair and a sense of integration. This, in turn, has enhanced their satisfaction with today’s society.

*“Community culture activities are very good. I attend all kind if activities, such as rope skipping, shuttlecock kicking etc. I am not a person who is willing to stay at home lonely.” (No. 7, born 1949)*

*“The community organizes activities including teaching people to use computer and knowledge about nutrition.” (No. 13, born1951)*

The narratives of our interviewees reveal a pervasive sentiment of satisfaction and contentment. These individuals have endured a multitude of challenges in the past, yet they have persevered and dedicated themselves to their country, society, and families amidst their struggles. In the present, they have benefited from a range of protections and services, which have enabled them to integrate their past experiences with their current circumstances. As a result, they feel a sense of fulfillment and joy.

#### 4.3.2 Joy and worry

Our interviewees have witnessed the development and changes of the times in their life course, and they tend to evaluate our society from the perspective of development and put forward their views with historical significance. As witnesses of wars and famines, they are happy about the prosperity of our society, but the changing society also raises some questions and concerns.

##### 4.3.2.1 Development, changes, and problems

In their narratives, we can discern the evolution and transformation of the country and society, which have become more prosperous and affluent. These changes have also bolstered the interviewees’ confidence and positivity. One individual stated, *“In my opinion, our society developed better and better. Now we are not afraid of being invaded by foreign countries because China has become strong. We will not be afraid anymore when we become strong.”* This old woman, born in 1938 (No. 16), experienced AJW and CW in her childhood, GF and CR in her youth. She now perceives China’s increasing strength and feels relieved and joyful because we no longer have to fear other countries.

From a social development perspective, our interviewees observed positive changes in society. Poverty, hunger, and chaos gradually disappeared, replaced by affluence and harmony. A version that directly reflects development is as follows:

*“After the founding of the People's Republic of China, people became richer gradually, but it was not possible to become richer very quickly…” (No. 11, born 1940)*

Development also came with problems. The country and society were no longer poor, and the economic boom bred problems of corruption. For interviewees who have experienced poverty and hunger, the embezzlement of people’s money is the last thing they want to see. A significant proportion of our interviewees expressed strong criticism and blame for corruption.

*“The corruption of government officials couldn’t be solved till now.” (No. 3, born 1948)*

*“Though it is better than before, but the gap is still very big. Corruption is also very serious.” (No. 4, born 1941)*

*“Our country now, corruption can’t be managed.” (No. 11, born 1940)*

The existence of hunger, poverty, and war in the past does not negate the existence of positive aspects of the past. Older adults did not lose their zest for life due to poverty and hunger; rather, they demonstrated resilience and solidarity in overcoming these challenges. *“My class director of primary school who treated me as same as my mother did. At that time, people treated each other with affection, not like today, affectional relation between people is quite weak, most relationships were built on money.”* This woman born in 1949 (No. 7) shows us a story full of human feelings and emotions.

Today, people no longer trust each other so much as before. Social development has brought about affluence, but it has also taken away the emotion of “mutual help.” *“Trust between people generally lowers, cohesion of society is not high, and there are also a lot of social problems. Such as food safety, in the past, fruits and vegetables can be eaten directly, which causes no problems, but it is not OK now (No. 4, born 1941).”* Contaminated food is served on people’s tables, and the problem of food safety is a typical manifestation of a crisis of confidence in society. This interviewee directly pointed out the correlation between the two.

Other interviewees were also concerned about food safety. *“Our country is seriously polluted now, there are no vegetable unpolluted, vegetables and food supplies are all polluted (No. 11, born 1940)”.*

Our interviewees were able to integrate the past and the present and to evaluate today’s society from a holistic perspective. A common theme among the interviewees was the joy of social development, accompanied by concerns about the potential consequences of this progress. The changes that have occurred in society, as well as the contrasts between the past and the present, have become an important criterion for their evaluation of society.

### 4.4 Expectations for youth: encouragement and inheritance for the next generation

Regarding the current young generation, our interviewees expressed uniformly positive expectations for them. The older adults focused their attention on the growth and success of the youth group, and were able to identify and assess the encounters and opportunities in the context of the new era in which the young generation is living. In reflecting on their own lives, the elders integrated their experiences and lessons, condensed their life wisdom, and hoped that the young generation would learn and pass on their wisdom, and continue to maintain their good style of hard work and endeavor.

#### 4.4.1 Youth in our time

Our interviewees look at the young generation from the perspective of the development of the era. Nowadays, the family and society can offer the younger generation more favorable conditions for living, education, and employment, which in turn allows them to achieve remarkable things. The opportunities of the times are as valuable as a vibrant youth, and the elders expressed their earnest expectations and encouragement.

##### 4.4.1.1 Hope, opportunities, and challenges

Many of our interviewees thought that they now lived in a prosperous, strong, open, and inclusive society that offers better conditions and opportunities for young people than the previous generation. This can be exemplified by the following direct version: *“You did not experience the hardship. You are so fortunate and blessed to be able to catch up with this era,”* stated an old woman born in 1938 (No. 12), whose father left home to join the army when she was born, and who was only able to graduate from junior high school before joining the workforce. This experience led her to believe that young people nowadays are catching up with a good era, as exemplified by the following: *“You are catching up with a good era now, how could girls be allowed to go to graduate school? (No. 12, born 1938)”*

The expectations of young people are related to their experiences and events of their own youth. Similar to the woman previously mentioned (No. 12), many of the interviewees expressed views based on inter-generational comparisons. They noted that in times past, people lacked access to basic necessities such as money, food, and education, as well as the right to choose their own work. *“Your young generation is insatiate; your life is much better than mine when I was a child. When I was as young as you are now, it was very difficult for me to have money to spend. There was little money or no money, no good food to be eaten (No. 1, born 1949)”.*

One of the older adults talked about the lack of autonomy in their lives, the inability to pursue their passions, and the lack of flexibility in their lifestyle. After graduating from university, he had to obey the assignment and was not free to choose his work and life. *“I was subject to appointment of government office when I finished my university education, I went to where I was asked to go and did what I was asked to do.” (No. 8, born 1938).* No. 8 posits that a greater number of free and selective young people should be able to identify their passions and pursue them with dedication, recognizing the value of the opportunities available to them and avoiding the waste of time. He asserts, *“You will not succeed if you do not work hard and find your way to make a living…”* This older adult’s account is a common narrative of the era (e.g., Nos. 4 and 16).

In addition to the comparison between the present and the past made from the perspective of the times, our interviewees also focused their attention on the youth group itself. The period of youth is precious, and people in their youthful years have vigorous physical strength and plenty of time. *“You have enough of time, how lucky you are still very young” (No. 2, born 1939).* Interviewees in their later years placed their hopes for building the country on the younger generation, one subject stated, *“I envy that you are so young. The future of our country is relying on your generation” (No. 10, born 1946).*

The contemporary era is the achievement of the collective efforts of the Chinese people, and the pursuit of a fulfilling life is a testament to their resilience. In this era of abundant opportunities and aspirations, the younger generation must appreciate the significance of this era and seize the opportunity to contribute to the advancement of the nation and society.

*“I have a request towards young people like you that you should know it is not easy to achieve today’s good life, don’t just intent to covet personal wealth; you should try your best to contribute more to the country.” (No. 11, born 1940)*

#### 4.4.2 Inheritance of wisdom

In considering the expectations for young people, our interviewees look back on their own lives and integrate past experiences, extracting from them the positive qualities that they or their contemporaries possess, and condensing the wisdom of their own lives. This wisdom is derived from the lessons of past lives, sublimated in suffering, and integrated into old age. They hope that the young generation can learn from their excellent character and style, and then pass on the wisdom of life. In addition, the interviewees identified the current challenges facing young people.

##### 4.4.2.1 Problems, expectations, and encouragement

Some of the interviewees discussed the perceived characteristics of the youth in their eyes. They identified several challenges facing young people, including a lack of resilience, an inability to withstand adversity, and a tendency to prioritize enjoyment over perseverance. One interviewee noted, *“The main problem is today’s young people are not surefooted. Another one is hedonism, today’s young people are unwilling to endure hardship.” (No. 8, born 1938)* This old man who experienced CW, GF, and CR, criticized his daughter for her hedonistic tendencies. He advocated that the spirit of hard work should be cultivated in young people, and believe that only through hard work can they ultimately achieve happiness.

Our interviewees had all experienced a life of hunger and poverty, and a lack of food and clothing were common memories. The poverty of their early lives prompted them to adopt frugal and thrifty living habits. Conversely, young people who have not experienced a period of material deprivation may lack a sense of thrift, which our interviewees expressed concern about.

*“I felt pity that many young people threw away boxes of apples and some other things which were still useful. I felt that other sides of today’s young people were OK, but they wasted too much.” (No. 5, born 1941)*

*“I hope that young generation would keep being frugal and hardworking.” (No. 14, born 1938)*

One of the older adults observed that younger individuals exhibited several deficiencies, including a lack of exercise, impatience, a tendency towards emotional volatility, a lack of patience, a lack of vocational skills, and a general lack of basic life skills. *“I feel that today’s young people are relatively lazy. Another point is that today’s young people are not surefooted, and are relatively impetuous. Still, another issue is that many young people just rely on their parents to make a living, do not try to find a job and make a living all by themselves.” (No. 4, born 1941)* In response to these deficiencies, the man advocates for young people to reflect on their circumstances and implement necessary changes, such as regular exercise, positive mental attitude, and spiritual growth. *“In my opinion, no matter how good today’s life condition is, young people should have some life skills… There are many issues that young people should think through, they should learn to reflect themselves”.*

From the narratives, it is evident that the expectations of older adults towards the younger generation are numerous. These include the importance of cherishing time, continuous self-improvement, striving for advancement, and making a meaningful contribution to the country and society. A straightforward version is: *“You should study hard to requite your parents and society”* (No. 12, born 1938).

From the narratives, it is evident that the conventional wisdom among older adults is that young people should prioritize the value of their time, pursue continuous self-improvement and contribute to their communities. One illustrative example is the following assertion: *“You should study hard to repay your parents and society for their investment in you” (No. 12, born 1938).*

A veteran (No. 10), born in 1946, who was wounded in the army and served as a village branch secretary for more than 10 years after he returned to his hometown after being discharged from the army, recounted his story by mentioning that: *“I felt a responsibility to be the oldest brother and to take good care of my younger siblings.”* As the eldest of his siblings, he felt a sense of responsibility and obligation. When discussing the encouragement and expectations he had for the younger generation, he highlighted that many young people who are only children lack exercise and have not experienced much hardship. *“However, the majority of you are the only child of your parents and have not experienced hardship, lack of discipline, or been spoiled by your parents. Consequently, you must develop good discipline. If you work hard and study hard, you will undoubtedly be promising in the future”.*

In discussing life lessons, an old woman born in 1939 (No. 2) posits that life is about experiencing suffering initially, followed by a period of sweetness. *“I realize that people are blessed later after they suffered in early time.”* She witnessed the loss of her loved ones and faced discrimination. Nevertheless, she maintains that life is about experiencing hardship initially and then being blessed later. *“I also asked my son this way, which was to study hard and get a high education, we have nothing to rely on, what we only own is our knowledge and skill.”* She urged her son to study hard and utilize her knowledge and skill. This is an illustrative example of an older individual imparting life wisdom to the younger generation.

### 4.5 Summary of key themes

In summary, the findings reveal that a dynamic interplay of individual experiences, societal transformations, and future-oriented expectations shapes the life wisdom and happiness of Chinese older adults. Personal well-being is grounded in the pursuit of education, family bonds, and active social participation. Their perceptions of society demonstrate a balance between contentment and critical reflection. Furthermore, their aspirations for the next generation emphasize resilience and the transmission of accumulated wisdom. These dimensions collectively illustrate a nuanced portrait of aging in contemporary China, as summarized in Table 2.

**Table 2:** Themes of life wisdom and happiness.

Topic	Dimensions	Themes
Life wisdom and happiness	Individuals	1. Education: valuing education
		2. The inter-connectivity between family members
		3. Active social participation
	Society	1. Contentment and gratitude to society
		2. Joy and worry: caring about society
	Future	1. Youth in our Time: caring about the next generation
		2. Inheritance of Wisdom

## 5 Conclusion and discussion

Based on the interview data, the study revealed that the themes of happiness and life wisdom among Chinese older adult are mainly centered on several aspects: First, they place a strong emphasis on education, maintain close relationships with their families, and actively engage in social activities, reflecting a close connection between personal well-being and interpersonal involvement. Second, they express satisfaction and gratitude for social welfare, while simultaneously experiencing both joy and concern regarding social development, illustrating their internalization and critical reflection on societal values. Finally, they hold high expectations for the younger generation, encouraging them and passing on their wisdom, thereby demonstrating their acceptance and recognition of their entire life experiences and deep emotional attachment to the youth.

The narratives of the interviewed older adults indicate that in contrast to the turbulence of their early lives (34), structure, continuity, and predictability are the dominant tones of their midlife and current lifestyles. Furthermore, the future trajectory of their lives remains within a predictable range. In Western countries, the oscillation between integration and despair in old age is often viewed as a process of harmonizing the life cycle. Consequently, the concept of VINDOA emerges as a crucial area for further investigation, particularly regarding how older adults perceive the individual, society, and the future, as well as how they narrate their experiences.

A significant finding is that the way older people view life has a profound impact on their personal experience and well-being in the present moment. This philosophy of life fosters self-compassion in old age and overcome the negativity that comes from their past and present life. According to Erikson et al. (64), the fundamental conflict in this stage arises from the tension between integration and despair. In this stage, older adults make every effort to integrate the various psychosocial themes of their lives, striving to achieve balance, while simultaneously facing the challenges brought by the proximity of death and the decline of physical functions. On the one hand, individuals strive to perpetuate their current state of happiness and thus achieve the integrity of their life course (62, 66). Conversely, as life draws to a close and physical functioning declines, feelings of exhaustion and inferiority emerge, creating a sense of being unable to keep up with the times. Additionally, fear and helplessness are negative emotions that older adults must overcome (67, 68). In many narratives, older adults prioritize their health, return to their families, establish new social networks, and embrace a slower, steadier pace of life. For some, such as No. 12 and No. 8, there is a discrepancy in their perceptions of their children, students, and other descendants, which in turn leads to the decision to be alone or to enter a relatively isolated life. These two opposing tensions became an important aspect of our respondents’ current life stage, finding a balance between a sense of persistent integration and a sense of fearful helplessness and despair. This process of integration can be regarded as an embodiment of wisdom, which emerges as individuals reconcile life experiences and achieve inner harmony, even in the face of inevitable decline (Erikson, 1963) (69, 70). At this stage, wisdom is not merely a matter of knowledge or cognitive ability, but more fundamentally involves emotional resilience and the capacity to adapt to the final phase of life. It enables individuals to find meaning in the aging process itself and to maintain the vital involvement in the necessary disinvolvements of old age.

Consciously and unconsciously, these older adults are also attempting to reconcile earlier psycho-social themes, including the dichotomy between reproduction and stagnation (71, 72), intimacy and loneliness (73, 74), and the tension of self-sameness and identity disorganization (34, 75), along with other earlier psychosocial themes, is reconciled through their integration with the current stage of old age. Having been verbally abused and beaten during the Cultural Revolution, No. 2 now confesses that he does not discriminate against those who hurt him. This is a philosophy of life that transforms the negative effects of a bitter experience into a motivation to live a positive life. It can be postulated that our interviewees did their utmost to integrate each psycho-social theme, including the memory of the structural breakdown brought about by the ephemeral breakdown, in an attempt to achieve a balance that would make the whole life cycle integrated.

Another finding was that to be able to make connections between the suffering past and the happy present, the groups studied had to be categorized within a more refined social stratification. Social stratification may play a crucial role in the dissolution of the negative impact of early life ephemeral breakdown on an individual’s happiness in later life. Older individuals from different social strata exhibit disparate comprehension of historical events, which is closely linked to their urban–rural backgrounds (76, 77), educational attainment (78, 79) and type of occupation (80, 81). Individuals in higher social classes tend to report a higher current quality of life. However, this does not imply that they did not necessarily avoid adverse experiences, such as war and famine, earlier in their lives.

No. 2 and No. 8 are both retired university professors. In No. 2’s account, she spent her childhood on the run and barely attended elementary school. No. 8 lost his mother in childhood and his family gave his biological sister away because they were poor. The upheavals experienced in childhood and youth serve as the impetus for class leaps and the current high quality of life, which is markedly different from the mainstream Western discussion of the development of the stages of the life course (82–84). The impact of education is evident in many of the stories. No. 7’s middle school studies were disrupted during CR, and she began working after her father’s death. After completing high school and college while working, her living conditions improved significantly. The early years of life also brought more than just painful memories; in the contrast between the past and the present, the informant expresses a fondness for the closer relationships and good social mores of the past.

Prolonged homebound status among retired older adults, coupled with a reduction in socialization and access to technological products, can result in feelings of disconnection or separation from their surroundings and activities in their social, family, and personal lives (85, 86). In contrast to the “despair of abandonment,” most of the older interviewed demonstrated contentment and optimism in their current lives. Rather than diminishing their passion for life, many became more proactive in forging connections with the community. This is a more desirable state of life in old age, as described by Erikson et al. (64). The motto of No. 3 is “A contented mind is perpetual feast,” and he often discusses with his children the lack of food in the past, which emphasizes the happiness of his current life. In old age, a sense of integration becomes the dominant harmonious tendency, balanced with a pervasive sense of despair. This culminates in a particular form of life wisdom. This life wisdom is rooted in mature hope, will, purpose, loyalty, love, and caring. Although physical and mental functions are declining, life wisdom dissolves despair more skillfully and integrates and transmits it to the next generation.

These narratives prompt us to consider the influence of social development on the lives of older individuals. China’s GDP has grown exponentially since the ROC, with a value that has exceeded 300 times its original value (87). Furthermore, as survivors of disasters such as wars and famines, interviewees expressed greater satisfaction with their current standard of living. The advancement of social development has had a positive impact on the well-being of older adults and has facilitated the realization of VINDOA and the harmonization of the life course. At the same time, as a society with a high degree of aging, the level of social participation of older people exerts a further influence on the direction of social development. If we shift our focus to what is occurring concurrently with universal modernization, we find that the value of suffering changes over time. No. 8 explicitly states that his children are not willing to suffer and tend to be hedonistic, suggesting that education on suffering history is still necessary for young people. The experience of suffering in early life is distilled into a significant component of the wisdom of old age. This wisdom not only has a positive impact on the well-being of the older adults but also continues to be transmitted to the next generation through verbal and nonverbal communication, thereby exerting a cultural influence.

## 6 Significance and need for further research

### 6.1 Significance

The study has a certain degree of significance. In terms of theoretical research, we present the life stories of Chinese older adults based on qualitative interview data, which enriches the life course theory and related research on happiness in old age. In terms of practical application, through the presentation of the tension between integration and despair, it provides a Chinese case study for understanding VINDOA and provides a reference for the study of the life course of groups experiencing continuous social change, development and transformation. Furthermore, the study advances our understanding of individual agency and demonstrates that resilience and wisdom in life can mitigate the negative impact of suffering. This, in turn, offers insights that can inform the development of gerontological social work.

### 6.2 Need for further research

The current study demonstrates the current stage of the Chinese older adults’ lifestyles and levels of well-being, which are embedded in older adults’ life wisdom and happiness. We have researched the life wisdom of the Chinese older adults, and further research is necessary to investigate how the understanding of life philosophy and well-being of older Chinese and Westerners is similar and different.

Our material illustrates the integration and fulfillment of the Chinese older adults’ life stories, but in fact, the disruption and fragmentation are also of interest. Whether older adults can self-heal from the “despair” in their lives by reviewing their life histories and telling their life stories offers the potential for new longitudinal research using narrative therapy and other methods.

## Data Availability

The original contributions presented in the study are included in the article/supplementary material, further inquiries can be directed to the corresponding authors.
